# Oxidative Stress and Autophagy: Unraveling the Hidden Threat to Boars’ Fertility

**DOI:** 10.3390/antiox14010002

**Published:** 2024-12-24

**Authors:** Ruizhi Hu, Xizi Yang, Jianhua He, Shusong Wu

**Affiliations:** 1 National Engineering Laboratory for Pollution Control and Waste Utilization in Livestock and Poultry Production, Institute of Subtropical Agriculture, The Chinese Academy of Sciences, Changsha 410125, China; 2 Hunan Collaborative Innovation Center for Utilization of Botanical Functional Ingredients, College of Animal Science and Technology, Hunan Agricultural University, Changsha 410128, China

**Keywords:** oxidative stress, autophagy, spermatogenesis, sperm quality

## Abstract

This review systematically examines the influence of oxidative stress on the reproductive function of male livestock, with a particular focus on the modulation of autophagy. Spermatogenesis, a highly precise biological process, is vulnerable to a range of internal and external factors, among which oxidative stress notably disrupts autophagic processes within the testes. This disruption results in diminished sperm quality, impaired testosterone synthesis, and compromised integrity of the blood–testis barrier. Furthermore, this review elucidates the molecular mechanisms by which oxidative stress-induced autophagy dysfunction impairs spermatogenesis and mitochondrial function, consequently reducing sperm motility. These findings aim to provide a theoretical foundation and serve as a reference for improving reproductive performance and sperm quality in livestock.

## 1. Introduction

**Figure 1:**
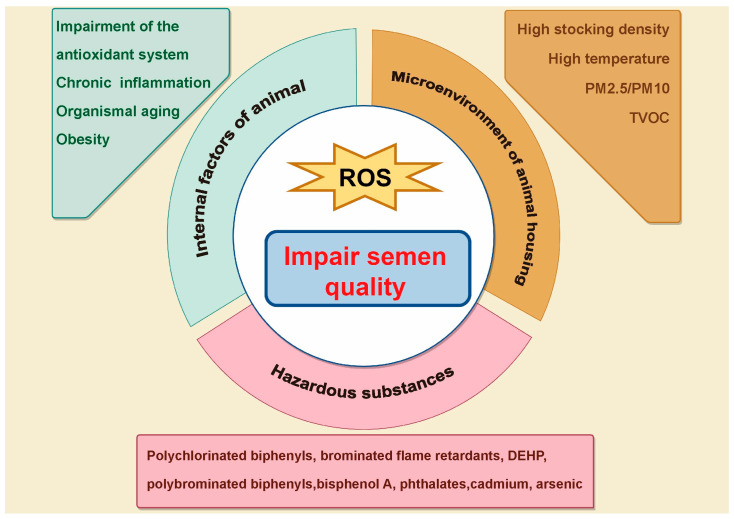
Factors leading to oxidative stress and autophagy impairment in livestock and poultry reproduction. Livestock production, inherent factors, suboptimal housing conditions, and exposure to toxic substances collectively contribute to oxidative stress via the generation of ROS. This oxidative stress subsequently disrupts autophagic mechanisms within the organism, resulting in compromised semen quality.

The reproductive function of male livestock is of considerable economic and productive importance within the livestock industry, as semen quality is a critical determinant of key performance metrics, including mating success rates and litter sizes. The generation of high-quality semen is essential for attaining efficient and high-yield breeding outcomes. This process relies not only on the proper functioning of the testes but also on the coordinated activity of other organs, including the epididymis, the hypothalamus–pituitary–testis axis, and the accessory sexual glands, as well as the intricate mechanisms involved in spermatogenesis. However, spermatogenesis is highly vulnerable to a range of internal and external disruptions, such as environmental conditions, pollutant exposure, and endogenous factors inherent to the animals. Interfering factors may induce oxidative stress within the body, a condition that occurs when the concentration of reactive oxygen species (ROS) surpasses the antioxidant defense system’s capacity to neutralize them, leading to a pathological state [1,2,3,4,5] (Figure 1). ROS are capable of directly damaging sperm DNA, proteins, and lipid membranes, and they also disrupt cellular signaling pathways, thereby further compromising testicular function and the process of spermatogenesis [6]. Numerous studies have demonstrated that exogenous antioxidant supplementation can significantly enhance semen quality by improving both sperm motility and count, thereby offering an effective strategy for addressing reproductive issues associated with oxidative stress [7,8,9].

Despite considerable advancements in this area, the exact mechanisms by which oxidative stress impacts semen quality are not yet fully elucidated. Recent research suggests that oxidative stress may indirectly regulate spermatogenesis and semen quality by influencing the homeostasis of autophagy [10,11]. Autophagy, a vital metabolic process for preserving cellular homeostasis, is crucial for spermatogenesis, sperm maturation, and the maintenance of sperm motility [12,13]. The dysregulation of autophagy induced by oxidative stress can result in compromised sperm production and reduced semen quality. Consequently, it is of substantial scientific and practical importance to further investigate the molecular mechanisms by which oxidative stress affects semen quality through autophagy. This review systematically investigates the potential mechanisms of action of ROS through autophagy in the regulation of spermatogenesis and sperm function. The objective is to offer novel insights for future research and to establish a theoretical foundation for developing intervention strategies aimed at enhancing male reproductive capacity.

## 2. Oxidative Stress and Autophagy

### 2.1. Autophagy

Autophagy is an evolutionarily conserved mechanism in eukaryotic cells, facilitating the degradation and recycling of intracellular macromolecules and damaged organelles. This process is typically categorized into three distinct types: macroautophagy, microautophagy, and chaperone-mediated autophagy. The earliest documented observation of autophagy pertained to macroautophagy. In 1956, Clark identified dense bodies with membrane-bound structures within cells while examining neonatal mouse kidney tissue using electron microscopy. These dense bodies frequently contained cytoplasmic structures resembling mitochondria [14]. At the 1963 International Symposium on Lysosomes, Christian de Duve characterized the process whereby membrane vesicles encapsulate cytoplasmic components and organelles within cells as autophagy. Contemporary research predominantly concentrates on macroautophagy, the mechanisms of which are the most comprehensively elucidated [15,16]. Macroautophagy is frequently, albeit somewhat reductively, synonymous with cellular autophagy. In contrast, microautophagy entails the direct sequestration of intracellular materials through the invagination of the lysosomal membrane, resulting in the formation of intralysosomal vesicles that subsequently release their contents into the lysosome for degradation [17]. Chaperone-mediated autophagy represents a specialized pathway for the degradation of proteins containing the KFERQ motif. Proteins with this motif are identified by the chaperone Heat shock cognate protein 70 (HSC70) and subsequently translocated into the lysosome through the Lysosomal-associated membrane protein 2A (LAMP2A) protein for degradation [18]. While the mechanisms underlying the three distinct forms of cellular autophagy differ, they collectively contribute significantly to the cellular response to external stimuli and the removal of damaged materials.

### 2.2. Autophagic Signaling

Cellular autophagy is meticulously regulated across all eukaryotic cells, with both deficiencies and excessive activity posing potential harm to the organism. The regulatory mechanisms governing autophagy are intricate, involving multiple intracellular signaling pathways, including the mammalian target of rapamycin (mTOR), AMP-activated protein kinase (AMPK), nuclear factor kappa-B (NF-κB), and hypoxia-inducible factor (HIF)-1α pathways [19]. The mTOR pathway, in particular, serves as a central signaling axis, orchestrating intracellular protein synthesis and degradation, energy metabolism, cell proliferation, apoptosis, and autophagy [20]. The mechanistic target of rapamycin (mTOR) exists in two distinct complexes: mTORC1 and mTORC2. mTORC1 is characterized by its sensitivity to rapamycin and its strong association with autophagy, primarily due to its adapter protein, Raptor. This complex is capable of responding to various external stimuli, including energy levels, amino acid availability, and stress conditions. Under physiological conditions, active mTORC1 phosphorylates downstream targets such as p70 ribosomal S6 kinase 1 (p70S6K1) and eukaryotic translation initiation factor 4E-binding protein 1 (4E-BP1), thereby facilitating protein synthesis. Concurrently, mTORC1 exerts a negative regulatory effect on autophagy by phosphorylating UNC51-like kinase-1 (ULK1) and autophagy related gene (Atg)13 [21].

AMPK functions as an upstream regulator of mTOR and is activated in response to cellular stress, particularly when intracellular ATP levels decrease [22]. Upon activation, AMPK inhibits mTOR activity, leading to a reduction in protein synthesis and cellular growth, while simultaneously promoting autophagy [22]. Under conditions of energy depletion or nutrient scarcity, AMPK is activated and undergoes phosphorylation to form p-AMPK. This phosphorylated form of AMPK modulates several autophagy-related proteins, including the activation of the autophagy-related ULK1 [23]. The activation of this pathway facilitates the expression of essential autophagy-related molecules, including LC3, p62, and Beclin1, which, in turn, enhances autophagosome formation [24]. Conversely, the mechanistic target of rapamycin mTOR, a downstream signaling component, acts in opposition to AMPK. Under conditions of high energy availability, mTOR becomes activated and phosphorylated (p-mTOR), leading to the inhibition of ULK1 activity. This inhibition impedes the formation of autophagic vesicles, thereby suppressing the autophagic process within the cell [25].

NF-κB and autophagy engage in bidirectional interactions within both physiological and pathological contexts [26]. The IKK/NF-κB signaling pathway modulates autophagy in a manner contingent upon specific stimuli. Empirical studies have shown that IKK/NF-κB can activate autophagy by directly upregulating the expression of genes or proteins integral to autophagosome formation, including Beclin1, the BAG3–HspB8 complex, ATG5, and LC3 [27]. In contrast, NF-κB can inhibit autophagy by upregulating the expression of autophagy suppressors, such as A20, members of the Bcl-2 family, PTEN/mTOR, and NO, or by downregulating the activity of autophagy inducers, including BNIP3, JNK1, p53, and ROS [28].

BNIP3 is a specific target gene of HIF-1α, which is fully expressed under conditions of moderate hypoxia [29]. Both BNIP3 and its homolog BNIP3L play roles in hypoxia-induced autophagy. Under moderate hypoxic conditions, HIF induces the expression of BNIP3 and disrupts the interaction between Beclin1 and Bcl-2. The release of free Beclin1 subsequently facilitates autophagy [30].

### 2.3. Mechanism of Autophagy Induced by Oxidative Stress

**Figure 2:**
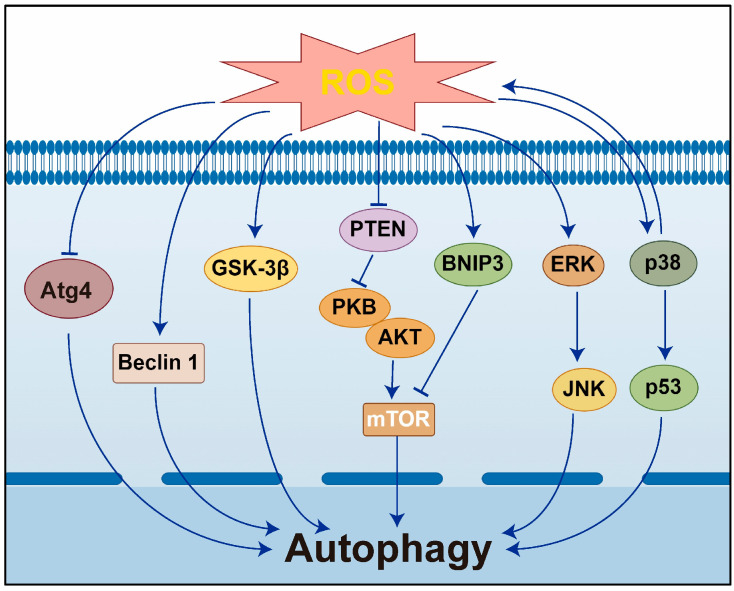
Mechanisms of autophagy induced by ROS. Under conditions of oxidative stress, ROS have the potential to induce autophagy through multiple pathways during the autophagosome formation process. ROS, reactive oxygen species; PTEN, phosphatase and tensin homolog; PKB, B protein kinase B; BNIP3, BCL2 interacting protein 3; mTOR, mammalian target of rapamycin; Atg4, autophagy-related protein 4; GSK-3β: glycogen synthase kinase 3β; ERK, extracellular signal-regulated kinase; JNK: c-Jun, c-Jun N-terminal kinase.

ROS are predominantly produced by the mitochondrial electron transport chain and various enzymatic reactions. Additionally, they can be triggered by external factors such as tumor necrosis factor-alpha (TNF-α) and lipopolysaccharides. Autophagy serves as a critical sensor of redox signaling within cellular responses. Under conditions of oxidative stress, ROS can initiate autophagy through multiple pathways involved in the autophagic process [31] (Figure 2). The autophagy-related proteins Atg7, Atg3, and Atg10 facilitate ubiquitin transfer through cysteine residues located at their catalytic sites. The thiol groups on these cysteine residues are notably vulnerable to oxidative modifications induced by ROS, suggesting that these proteins may be responsive to redox signals. Furthermore, the activity of kinases and phosphatases can be modulated by redox-mediated post-translational modifications. Such redox-dependent alterations of intermediates within signaling pathways can result in modifications to their activity, localization, or substrate specificity [32]. Under conditions of starvation, the elevated levels of intracellular H_2_O_2_ inhibit the activity of the Atg4 protease, thereby preventing the de-lipidation of LC3-II and facilitating the formation of autophagosomes [33]. Several studies have demonstrated that ROS can induce autophagy through mechanisms such as the disruption of mitochondrial membrane potential, inhibition of the Akt–mTOR signaling pathway, or activation of the Bcl-2-adenovirus E1B 19 kDa protein–interacting protein 3 (BNIP3)–mTOR pathway [32]. Furthermore, mitogen-activated protein kinases (MAPKs) have been identified as downstream effectors of ROS in the induction of autophagy. Specifically, ROS can activate extracellular signal-regulated kinases (ERKs) and c-Jun N-terminal kinases (JNKs), thereby promoting autophagy [34]. In A357 cells, mannose-binding lectin has been shown to induce autophagy through the mitochondrial-dependent ROS–p38–p53 signaling pathway [35]. ROS are also capable of inhibiting the phosphatase and tensin homolog (PTEN) pathway, consequently activating autophagy via the protein kinase B (PKB)–Akt pathway. Additionally, ROS can modulate calcium ion channels, leading to elevated intracellular calcium levels, which further promote autophagy [36].

## 3. Autophagy Is Involved in Spermatogenesis

Spermatogenesis is a meticulously orchestrated process occurring within the seminiferous epithelium, which can be delineated into three distinct stages (Table 1): (a) the self-renewal and differentiation of spermatogonia into spermatocytes; (b) the generation of round spermatids via the meiotic division of spermatocytes; and (c) the round spermatids undergo metamorphose into spermatozoa (including chromatin condensation via histone-to-protamine replacement, nuclear reshaping into a streamlined structure, acrosome formation from Golgi-derived vesicles containing hydrolytic enzymes, flagellum development from centriolar structures, redistribution of mitochondria around the flagellar axoneme, and cytoplasm reduction with residual bodies phagocytosed by Sertoli cells). Beyond the involvement of germ cells, somatic cells within the testes, including Leydig and Sertoli cells, play a crucial role in facilitating this process by establishing a conducive spermatogenic microenvironment. Autophagy is a critical mechanism for maintaining cellular homeostasis and is instrumental in regulating various physiological processes, including spermatogenesis. It influences spermatogenesis by participating in key aspects such as germ cell meiosis, spermiogenesis, the integrity of the blood–testis barrier, and the synthesis of testosterone.

### 3.1. Oxidative Stress Inhibits Autophagy and Causes Spermatogonial Cell Damage

Spermatogonial stem cells, despite constituting merely 1/5000 of the overall testicular cell population, are critical, as their loss or damage can lead to irreversible conditions such as oligozoospermia, asthenozoospermia, and even azoospermia [22]. Under normal physiological conditions, autophagy is present in spermatogonial stem cells, albeit at a low level of activity. Its principal function is to remove misfolded proteins and damaged organelles under stress, thereby optimizing the distribution of intracellular materials and energy and preserving the viability of spermatogonial cells [23]. The elevated rate of cellular division observed during spermatogenesis results in a concomitant increase in mitochondrial oxygen consumption and ROS production within spermatogonial cells. ROS are instrumental in initiating autophagy through the activation of various downstream signaling pathways. The regulation of testicular temperature is of paramount importance, with the optimal thermal range for boars being 17 °C to 21 °C. Heat stress in boars may occur when the average humidity in pig housing reaches 30% and the ambient temperature rises to 28 °C [24]. Adult boars generally possess a body mass of around 300 kg [26]; when subjected to environmental temperatures surpassing 32 °C for extended durations, boars of this mass are prone to heat stress, which consequently diminishes semen quality [27]. During such heat stress conditions, there is an elevation in testicular temperature, impairing thermoregulation and leading to increased levels of ROS within the testes, thereby disrupting oxidative homeostasis [28]. The elevation in ROS levels induced by heat stress results in the downregulation of autophagic activity in spermatogonial cells, thereby hindering the efficient degradation of damaged organelles such as mitochondria and the endoplasmic reticulum via autophagic pathways, ultimately diminishing cellular viability [39]. However, upon administration of antioxidant treatment, autophagy is reactivated through the AMPK/mTOR signaling pathway. This reactivation leads to an increase in autophagic vacuoles within the cytoplasm, which facilitate the sequestration and degradation of damaged organelles. Furthermore, an imbalance in the antioxidant system within the testes may result in aberrant autophagy. Specifically, a deficiency in glutathione leads to the persistent accumulation of LC3-II in spermatogonial cells and an increase in autophagic vacuoles, culminating in heightened cell death [25]. Additionally, mTOR is not only integral to the regulation of autophagy signaling pathways but also functions as a crucial signal for the proliferation and differentiation of spermatogonial cells. In experimental studies involving animals, the suppression of mTOR gene expression in mice was observed to diminish the differentiation potential of spermatogonial stem cells into spermatogonia as well as the subsequent differentiation of spermatogonia into spermatocytes. This finding implies that autophagy may regulate the further differentiation of spermatogonia by degrading essential proteins involved in cellular differentiation processes [29,30].

### 3.2. Oxidative Stress Disrupts Autophagy and Impairs Spermatogenesis

Spermatogenesis represents a complex and highly organized sequence of cellular differentiation, necessitating the restructuring of cellular architecture and the re-regulation of physiological functions. The efficient removal of cytoplasm is deemed essential for the generation of functional spermatozoa. Dysfunction in sperm is predominantly attributed to anomalies in the sperm head or flagellum. Autophagy is intricately associated with the process of spermiogenesis, and its absence results in abnormalities in both the sperm head and tail. In comparison with diploid germ cells, the expression levels of autophagy-related proteins, including LC3 and Atg7, are markedly elevated in elongating spermatids [47]. The reproductive cell-specific knockout of Atg7, which abrogates autophagy, leads to reduced testicular weight, sperm abnormalities, and a significant decline in fertility in male mice [45].

The acrosome, a specialized membrane-bound organelle situated at the anterior region of the sperm nucleus, plays a critical role in cumulus cell dispersion and the zona pellucida reaction during fertilization. The formation of the acrosome necessitates cytoskeletal reprogramming, which requires the induction of autophagy to facilitate cytoskeletal rearrangement. This process advances through four distinct stages: the Golgi phase, cap phase, acrosome phase, and maturation phase [48]. Autophagy is involved in acrosome formation starting from the Golgi phase. Under typical conditions, proacrosomal vesicles originating from the Golgi apparatus coalesce into a singular acrosomal vesicle at the terminus of the spermatid nucleus. Upon knockout of the Atg7 gene, the small vesicles originating from the Golgi apparatus are unable to fuse, culminating in the formation of multiple acrosomal vesicle structures. During the cap phase, the aggregation of these vesicles or the aggregates derived from the Golgi apparatus results in acrosome contraction, thereby inducing acrosomal malformations [47].

The evidence indicates that acrosomal malformations are likely attributable to the improper fusion and migration of proacrosomal vesicles to the nuclear region. Mechanistically, Atg7’s involvement in acrosome biogenesis is probably associated with its role in the induction of autophagy. During autophagy, LC3 primarily facilitates the lipid fusion of autophagic vesicles. In spermatids, LC3 colocalizes with Trans-Golgi Network 38 (TGN38) rather than with the acrosomal marker Sperm Protein 56 (SP56). Therefore, membrane-associated LC3 is potentially implicated in the vesicle fusion process of Golgi-derived proacrosomal vesicles and their subsequent transport to the acrosome. In the absence of Atg7, LC3 does not colocalize with TGN38, resulting in the accumulation of proacrosomal vesicles in the recessed region adjacent to the Golgi apparatus [48]. This accumulation ultimately disrupts the increase in acrosome volume during the later developmental stages, leading to defects in acrosome formation.

## 4. Oxidative Stress Impairs Autophagy and Disrupts the Testicular Spermatogenic Microenvironment

### 4.1. Oxidative Stress-Induced Autophagy Suppression Disrupts Sertoli Cell Function and Compromises the Blood–Testis Barrier

Sertoli cells represent the exclusive somatic cell type located within the seminiferous tubules of the testes in male animals. They establish direct interactions with germ cells and are pivotal in the regulation of spermatogenesis. Their functions encompass providing structural and nutritional support to developing germ cells, phagocytizing apoptotic germ cells, secreting growth factors, and modulating the differentiation and self-renewal of spermatogonial stem cells [49]. Growing evidence suggests that autophagy is actively occurring in Sertoli cells [50,51], contributing to the formation of ectoplasmic specializations, enhancing Sertoli–germ cell communication, and playing a vital role in maintaining testicular homeostasis [45].

As the sole somatic cells in direct contact with germ cells within the seminiferous epithelium, Sertoli cells establish a conducive microenvironment for germ cell development, offering both nutritional and structural support to the germ cells throughout the various stages of spermatogenesis. The blood–testis barrier, predominantly constituted by tight junctions, cytoplasmic specializations, desmosomes, and gap junctions among Sertoli cells, forms the cornerstone of the spermatogenic microenvironment. This barrier performs a dual role: it shields intraluminal germ cells from immune system detection while simultaneously supplying nutrients crucial for effective spermatogenesis [52]. In livestock, the integrity of the blood–testis barrier is critical for sustaining a stable spermatogenic milieu. Disruption of this barrier is associated with the onset of spermatogenic disorders in species such as bulls, boars, and roosters [53,54,55].

Autophagy is crucial for preserving the integrity of the blood–testis barrier. Research utilizing aging rat models has demonstrated that the blood–testis barrier undergoes damage characterized by reduced autophagic activity compared with normal controls. The activation of autophagy signaling pathways leads to the upregulation of related proteins, including LC3 and ATG5; proteins associated with tight junctions, such as Zonula Occludens-1 and Claudin-11; and proteins associated with cytoplasmic specialization, such as N-cadherin, to varying extents [56]. These findings suggest that the restoration of autophagy levels may reverse damage to the blood–testis barrier associated with aging, thereby enhancing sperm quality and parameters. In aging boars, the reestablishment of the blood–testis barrier has been shown to improve sperm count [53].

### 4.2. Oxidative Stress-Mediated Autophagy Dysfunction Impairs Leydig Cell Function and Testosterone Synthesis

Interstitial cells, which are prevalent in the testicular interstitium, serve as the primary source of steroid hormones. Through the synthesis and secretion of testosterone, these cells interact with Sertoli cells via androgen receptors, thereby offering nutritional and metabolic support as well as structural protection to spermatogonia and sperm. This interaction is crucial for the maintenance of normal spermatogenesis [57]. In boars exhibiting reduced testosterone secretion, germ cell abnormalities manifest within the testes, leading to the inhibition of spermatogenesis [58]. The incidence of autophagy in interstitial cells is notably higher compared with many other cell types, with autophagosomes frequently observed engulfing organelles, predominantly mitochondria, which are crucial for androgen synthesis [59]. The autophagic process is closely linked to testosterone production in interstitial cells, and deficiencies in autophagy are frequently associated with disruptions in testicular homeostasis [60].

In contemporary boar breeding practices, the effective breeding lifespan of boars has diminished from over 30 months in the 1990s to approximately 20 months at present. Our previous research has identified that this reduction is primarily attributable to decreased testosterone levels, which consequently result in a lower sperm count [61]. Furthermore, sperm count and motility are markedly reduced in aging boars compared with their younger counterparts. This decline is associated with decreased levels of antioxidant enzymes such as superoxide dismutase (SOD) and catalase (CAT) alongside elevated levels of malondialdehyde (MDA) in the testes of older boars, suggesting oxidative damage [53]. The deterioration of the antioxidant system within the testes results in elevated levels of ROS, subsequently inhibiting autophagy. Experimental treatment of interstitial cells from rats of varying ages with both autophagy inhibitors and activators demonstrated that exposure to autophagy inhibitors led to a reduction in the expression of steroid acute regulatory protein (StAR) and a decrease in testosterone production, accompanied by an increase in intracellular ROS levels. Conversely, treatment with autophagy activators was found to enhance steroidogenesis in the interstitial cells of aging rats and to decrease intracellular ROS levels. The deletion of the autophagy-related gene Beclin1 in the interstitial cells of aging rats resulted in diminished StAR expression and testosterone production alongside elevated levels of ROS [60]. These observations imply that the inhibition of autophagy in aging boars could play a role in the disruption of testosterone synthesis within interstitial cells.

In the context of testosterone biosynthesis, autophagy in interstitial cells is crucial not only for sustaining the activity of steroidogenic enzymes but also for facilitating cholesterol uptake and providing the necessary substrates for testosterone production [13,62]. Cholesterol serves as a precursor in the biosynthesis of testosterone within testicular interstitial cells [58]. The primary sources of cholesterol in these interstitial cells are extracellular transport and the conversion of lipid droplets within the cells [63]. In particular, extracellular low-density lipoprotein (LDL) is internalized via LDL receptors (LDLRs) and subsequently hydrolyzed into cholesterol within lysosomes through the action of proprotein convertase subtilisin/kexin type 9 (PCSK9) [64]. Apolipoprotein A-1 (Apo A-1) plays a role in the regulation of testosterone synthesis in testicular interstitial cells by utilizing extracellular high-density lipoprotein (HDL) [65]. HDL is internalized via the scavenger receptor class B type 1 (SR-BI), thereby supplying essential substrates for testosterone biosynthesis. Inhibition of SR-BI expression results in inadequate intracellular cholesterol availability, consequently diminishing testosterone production [66]. These findings imply that HDL may exert an influence on testosterone synthesis. In interstitial cells, lipid droplets undergo degradation through lipolysis-related enzymes such as PLIN1, HSL, and ATGL, resulting in their conversion into smaller components and subsequent transformation into cholesterol, which is then distributed throughout the cytoplasm [67]. The inhibition of autophagy leads to the accumulation of lipid droplets within testicular interstitial cells, a reduction in free cholesterol content, and a subsequent decrease in testosterone synthesis [68]. NHERF2, which acts as a negative regulator of SR-BI, is degraded via the autophagy–lysosome pathway in these cells. The deletion of Atg5 or Atg7 leads to an abnormal accumulation of NHERF2, which subsequently downregulates SR-BI, resulting in inadequate cholesterol uptake and a consequent reduction in testosterone biosynthesis [66]. Testicular autophagy and serum testosterone levels increase from 4 to 18 weeks post-birth, with testosterone synthesis in developing boars being regulated by SIRT1. SIRT1 facilitates the degradation of NHERF2 through autophagy, thereby diminishing its inhibitory effect on the HDL receptor SR-BI in Leydig cells [69].

The inhibition of mitophagy induced by ROS can disrupt the ultimate synthesis of testosterone. Upon entering the mitochondrial matrix, cholesterol undergoes conversion to pregnenolone via cytochrome P450scc, followed by sequential conversion to testosterone through the enzymatic actions of 3β-HSD and CYP1737. In interstitial cells, increased ROS levels result in autophagy deficiency, which is associated with decreased levels of testosterone and StAR protein as well as mitochondrial dysfunction. The administration of the autophagy inducer rapamycin reinstates StAR expression and testosterone synthesis in interstitial cells whose function has been inhibited by ROS. This observation suggests that a reduction in autophagic activity may result in the accumulation of ROS, thereby impairing steroidogenesis in these cells [13].

## 5. Oxidative Stress-Induced Mitophagy Inhibition Leads to Reduced Sperm Viability

Sperm motility is intrinsically linked to fertility rates in livestock, as only sperm exhibiting normal forward motility are capable of reaching the ampulla of the fallopian tube to fertilize the oocyte. The movement of the sperm tail is dependent on ATP, with the mitochondria serving as a primary site for ATP synthesis. Alterations in the mitochondrial structure within sperm are frequently associated with modifications in energy metabolism enzymes, which can result in motility disorders [70]. Throughout spermatogenesis, mitochondrial morphology undergoes significant dynamic changes. Spermatogonia and early spermatocytes possess conventional mitochondria, whereas late spermatocytes, spermatids, and spermatozoa exhibit mitochondria that are highly condensed, reflecting enhanced metabolic efficiency at this developmental stage [71]. In mature spermatozoa, the number of mitochondria ranges from 22 to 75, and these organelles are interconnected, forming a compact helical structure around the midpiece of the flagellum [72]. Structurally, mitochondria are primarily composed of the outer membrane, inner membrane, intermembrane space, and matrix. The inner mitochondrial membrane undergoes folding to form cristae, which house the enzyme complexes of the respiratory chain and ATP synthase. These structures are integral to electron transport within the respiratory chain and facilitate the creation of a transmembrane proton gradient essential for ATP synthesis, catalyzed by ATP synthase [73]. Sperm motility is dependent on the energy supplied by the mitochondria. Alterations in mitochondrial architecture are linked to changes in various enzymes involved in energy metabolism, including cytochrome oxidase, succinate dehydrogenase, and lactate dehydrogenase isoenzymes. These changes can impact the energy supply and result in sperm motility disorders [74].

Upon ROS stimulation, mitochondria may experience depolarization-induced damage. To remove these impaired mitochondria, cells initiate a specialized autophagic process known as mitophagy, which specifically targets and degrades the dysfunctional organelles. The molecular mechanisms underlying mitophagy are intricate. In yeast, mitophagy predominantly depends on the regulation of autophagy-related genes. When mitochondria sustain damage, ATG32, located on the outer mitochondrial membrane, undergoes phosphorylation and activation. This leads to the formation of an ATG32–ATG11 complex, which interacts with ATG8 to facilitate autophagosome extension, thereby recruiting the autophagic machinery [75]. The disruption of acrosome formation in mouse germ cells and mitochondrial rearrangement upon the knockout of ATG5 and ATG7 genes indicates the critical role of mitophagy in acrosome development and the reorganization of mitochondria within the sperm tail [23,76]. Importantly, mitochondria serve as a principal source of ROS. During oxidative phosphorylation, mitochondria predominantly produce O_2_^•−^ in sperm, which can subsequently be converted into H_2_O_2_ through dismutation reactions [77]. Spermatozoa possess two distinct ROS-generating systems: the NADPH oxidase located on the sperm membrane and the NADH system situated in the sperm midpiece, which is associated with the mitochondrial respiratory chain [78]. Empirical evidence suggests that low concentrations of ROS are essential for several critical processes, including sperm maturation, capacitation, the acrosome reaction, binding to the zona pellucida, and fertilization of the oocyte. Furthermore, ROS-induced lipid peroxidation is known to enhance zona pellucida binding and to modulate the phosphorylation–dephosphorylation of sperm protein tyrosine residues [79]. Our research group determined that boar sperm with low motility demonstrated elevated ROS levels accompanied by disordered and abnormal mitochondrial organization within the flagellar sheath [35]. Recent studies indicate a negative correlation between seminal ROS levels and the oxidative DNA product 8-hydroxy-2′-deoxyguanosine (8-OHdG) with progressive sperm motility, while a positive correlation exists between seminal ROS levels and sperm 8-OHdG concentration. These findings imply that mitochondrial dysfunction induced by excessive ROS is a significant factor contributing to diminished sperm motility [80].

Mitophagy is predominantly governed by the PINK1/Parkin signaling pathway. PINK1, a protein located on the mitochondrial outer membrane, possesses serine/threonine kinase activity and functions as a sensor for mitochondrial damage [81]. Parkin, encoded by the PARK2 gene, is an E3 ubiquitin ligase responsible for mediating substrate ubiquitination and regulating protein degradation [81]. Both PINK1 and Parkin are ubiquitously expressed across a variety of tissues and organs, and their interaction facilitates mitophagy by directing damaged mitochondria toward degradation through the autophagy–lysosome pathway [82]. Under physiological conditions, PINK1 is expressed at minimal levels and undergoes rapid degradation upon its translocation into the mitochondria. In response to oxidative stress, PINK1 detects a reduction in mitochondrial membrane potential, leading to its stabilization on the outer mitochondrial membrane, where it recruits Parkin to initiate the process of mitophagy [82,83]. Parkin subsequently ubiquitinates substrates including VDAC1, Mfn1, and Mfn2, thereby modulating mitochondrial morphology. The ubiquitinated mitochondria are subsequently targeted to autophagic vacuoles through the mediation of autophagy receptor proteins such as NDP52, OPTN, p62, and LC3, culminating in the formation of mitophagosomes [83]. These mitophagosomes then proceed to fuse with lysosomes, completing the degradation process.

AMPK is capable of mediating the phosphorylation of the autophagy initiator ULK1, thereby promoting its translocation to the mitochondria [84]. Once activated, ULK1 interacts with various mitochondrial autophagy-related proteins, including BCL2/adenovirus E1 B-19kDa-interacting protein 3, FUN14 domain-containing protein 1, and BCL2-like protein 13. This interaction enhances their association with LC3, thereby facilitating the process of mitophagy [85]. AMPK serves as a positive regulator of autophagy and additionally modulates mitochondrial biogenesis via the deacetylation of the transcription factor peroxisome proliferator-activated receptor-gamma coactivator 1-alpha (PGC-1α) [86]. It also regulates mitophagy through the SIRT1–PGC-1α pathway, as demonstrated by Yi et al. [87]. Our study revealed that the activation of AMPK enhances PGC-1α expression, restores mitochondrial morphology, and improves boar sperm motility [35,61].

## 6. Perspectives

The modulation of nutrition presents considerable potential for enhancing autophagic activity and, subsequently, semen quality. Dietary supplements, including antioxidants such as vitamin E, selenium, and polyphenols, have the capacity to mitigate oxidative stress and augment autophagic flux. Furthermore, nutrients that affect critical signaling pathways, such as AMPK/mTOR, may provide further advantages. Research indicates that diets with 15% and 30% energy restriction trigger testicular autophagy and apoptosis through the activation of the AMPK–ULK1 signaling pathway [88]. This process involves the upregulation of Beclin-1, the LC3-II/LC3-I ratio, and the activation of AMPK, p-AMPK, and ULK1 [88]. Additionally, folic acid supplementation in feed has been observed to enhance the expression levels of Beclin1 and ATG5 proteins in testicular tissue, thereby promoting autophagy and improving semen quality [89]. Furthermore, the incorporation of metformin into the diet markedly upregulates the expression of Beclin1 and LC3 proteins, concurrently enhancing sperm motility, membrane functionality, and acrosome integrity [90]. The aforementioned study indicates that the nutritional regulation of autophagy offers a promising strategy for enhancing semen quality in future applications.

## 7. Conclusions

**Figure 3:**
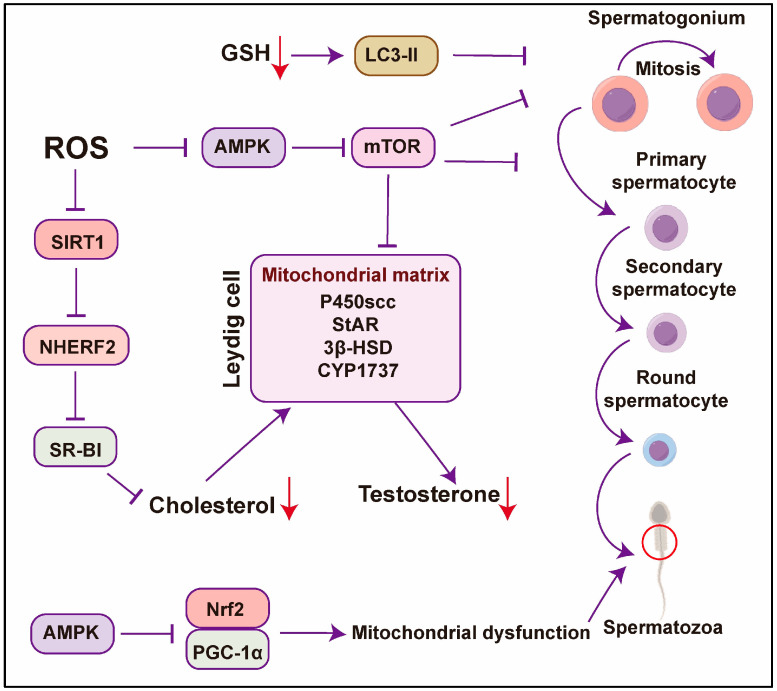
Mechanisms of oxidative stress-mediated autophagy in male livestock reproduction. ROS inhibits autophagy and disrupts mitochondrial function, leading to reduced cholesterol metabolism and testosterone synthesis, which subsequently impairs different stages of spermatogenesis. Red circle, sperm mitochondria. AMPK promotes autophagy and protects mitochondrial integrity, alleviating oxidative damage, while excessive mTOR activation suppresses autophagy. Ultimately, autophagy deficiency and mitochondrial dysfunction result in decreased testosterone levels and abnormalities in sperm maturation.

Autophagy is integral to the maintenance of male reproductive health, particularly in the context of spermatogenesis. It is essential for the removal of damaged organelles, cytoplasmic remodeling, and the regulation of spermatogonia stem cell activity, in addition to supporting the functions of the Sertoli and Leydig cells. Oxidative stress significantly disrupts autophagic activity, resulting in compromised spermatogenesis, testosterone synthesis, and sperm quality. The restoration of autophagic function via targeted interventions, including antioxidant treatments or modulation of the AMPK/mTOR pathway, has the potential to ameliorate these impairments (Figure 3). Furthermore, the regulation of autophagy presents a novel approach to enhancing reproductive performance in livestock. Continued research is imperative to elucidate the molecular mechanisms underpinning autophagy and to develop therapeutic strategies that leverage its regulatory roles within reproductive biology.

## Figures and Tables

**Table 1:** Effect of autophagy on spermatogenesis.

Testicular Cell Types	Effects of Autophagy	Result	References
Leydig cell	Maintain the testosterone synthesis.	Autophagy downregulation in interstitial cells reduces NHERF2 degradation via the autophagy–lysosomal pathway, leading to decreased steroid hormone synthesis.	[37]
		In aged Beclin1 knockout rats, stromal cells show reduced StAR expression and testosterone production.	[13]
Sertoli cell	Maintain the microenvironment of spermatogenic progression.	Knockout of Atg7 or Atg5 disrupts apical ectoplasmic specialization in the spermatogenic epithelium, compromising the blood–testis barrier integrity.	[38]
Spermatogonia	Maintenance activity of spermatogonia.	AMPK/mTOR pathway activation promotes autophagy, aiding in the clearance of damaged organelles and supporting spermatogonia activity.	[25,39]
Spermatocyte	Maintain meiosis.	Autophagy initiation enhances phagocytic removal of meiotic spermatocyte residues, supporting cellular division.	[40]
		Atg7-mediated autophagy reduces heat stress-induced spermatocyte apoptosis.	[41]
Spermatid	Maintain the formation of normal spermatozoa.	Silencing Beclin1 in Leydig or germ cells suppresses autophagy, leading to Golgi-derived acrosomal vesicle formation and acrosomal defects.	[42]
		Selective inhibition of autophagic flux in spermatozoa causes ribosomal subunit accumulation in the cytoplasm, mitochondrial ridge vacuolization, and signs of mitochondrial dysfunction.	[12]
		LC3 and ATG7 expression is significantly upregulated during the transition from round to elongated sperm.	[43]
		Disruption of LC3 and ATG7 acetylation hinders spermatogenesis.	[44]
		Targeted Atg7 knockout in germ cells, eliminating autophagy, reduces testicular weight and increases sperm malformations.	[45]
		Autophagy suppression leads to PDLIM1 accumulation, impairing cytoplasmic clearance and compromising sperm flagellar structure integrity.	[46]

## Data Availability

Not applicable.
